# Analysis on the Influence Path of User Knowledge Withholding in Virtual Academic Community – Based on Structural Equation Method-Artificial Neural Network Model

**DOI:** 10.3389/fpsyg.2022.764857

**Published:** 2022-02-07

**Authors:** Chengyi Le, Wenxin Li

**Affiliations:** School of Economics and Management, East China Jiaotong University, Nanchang, China

**Keywords:** virtual academic community, knowledge withholding intention, knowledge hiding, artificial neural network (ANN), SEM-ANN

## Abstract

The phenomenon of knowledge withholding is a vital issue that undermines knowledge sharing and innovation, hinders the development of offline and online organizations. Clarifying the relationship between influencing factors and knowledge withholding is significant to improve the phenomenon of knowledge withholding in offline and online organizations. Few types of research focus on the online virtual academic community and integrate the three factors of knowledge, individual, and environment to research knowledge withholding. To solve the limitation, this research is based on sociology and psychology-related theories. The two dimensions of enabling and inhibition are divided into factors affecting knowledge withholding. An attempt is made to explore the path between the three types of factors influencing knowledge, individual and environment, and knowledge withholding. This study collected data from 616 users in China’s virtual academic community. It used a structural equation model combined with a cross-layer connected neural network to conduct an empirical analysis on the proposed hypothesis. The results found that: in the virtual academic community, knowledge power in the enabling dimension is the main reason for users to form knowledge psychological ownership, which affects users’ knowledge withholding. However, the effect of professional commitment on users’ knowledge psychological ownership is not significant. After SEM-ANN model fitting, the combined inhibitory effect of community privacy protection and community reciprocity on user knowledge withholding in the inhibition dimension is significantly improved. This research has a specific guiding significance for enhancing the knowledge withholding phenomenon of the virtual academic community and creating an excellent academic exchange atmosphere.

## Introduction

At the end of the 20th century, the organization for Economic Cooperation and Development first put forward the concept of ‘knowledge economy.’ At present, in the rapid development of information technology, the knowledge economy has played an essential role in social progress and complements information technology. With the improvement of information technology and internet, the emergence of virtual communities breaks the restrictions of time and space. Further, it opens up a new way for the dissemination of social information and knowledge. This kind of community is a new social space supported by the internet. The rapidly developing virtual community also provides a more convenient channel for scientific research cooperation. This virtual community, which relies on the network and gathers all kinds of researchers and scholars, is called virtual academic community. Such communities have realized cross regional, efficient and large-scale scientific research cooperation among users. Nowadays, with the maturity and improvement of virtual academic communities such as ResearchGate, Academia, Xiaomuchong, Jingguanzhijia, Dingxiangyuan etc., virtual academic communities have gradually become mainstream scientific research platforms (Liu et al., 2020). Efficient knowledge sharing is an essential support for the continuous innovation and sustainable development of scientific researchers. More and more scientific research users choose to join the virtual academic community to learn about subject development and cutting-edge information through timely knowledge sharing, and then achieve collective and personal goals (Hui and Pritchard, 2014; Tan and Li, 2020).

However, surveys show that when facing knowledge sharing, 46% of the Chinese sample respondents admit that they have hidden knowledge (Peng, 2013), 76% of respondents in the US sample indicated that they actively or passively retained their knowledge (Babcock, 2004). This phenomenon is widespread in the virtual community, a community for communication and knowledge sharing (Shen et al., 2019). In the user group of the virtual community, 90% of users are diving, 9% of users occasionally contribute knowledge, and only 1% of users contribute most of the knowledge of the community (Antelmi et al., 2019). The research shows that there is a large amount of knowledge information in the virtual academic community, it provides users with a convenient communication space. However, there are many problems in the community, such as many diving users, low awareness, low knowledge utilization, low knowledge contribution and willingness to participate (He et al., 2021). This kind of knowledge withholding behavior seriously hinders the knowledge flow of the virtual academic community, undermines the knowledge creation of the virtual academic community, and reduces the influence of the virtual academic community (Cerne et al., 2014; Bogilović et al., 2017). In this case, how to minimize users’ willingness to retain knowledge in the community and promote user knowledge sharing has become a new problem that needs to be solved urgently.

Knowledge withholding originated from organizational behavior, manifested as the individual’s contribution to knowledge is less than its maximum contribution ability (Lin and Huang, 2010). As a collection of possible behaviors, knowledge withholding includes knowledge hiding and hoarding. Knowledge hiding reflects the conscious concealing behavior of individuals, and knowledge hoarding leads to the unconscious accumulation of knowledge (Connelly et al., 2012). Combining the above viewpoints, this article defines knowledge withholding as a broad concept, covering knowledge hiding, knowledge hoarding, and other anti-knowledge production behaviors. The existing literature on knowledge withholding mainly focuses on the two areas of offline entity organizations and online virtual communities. The research on employee knowledge withholding within offline entity organizations has the earliest origin and the most fruitful research results.

In offline entity organization research, scholars mainly focus on knowledge attributes, personal factors, and environmental atmosphere. (1) The natural attribute of knowledge is one of the primary prerequisites generally recognized by scholars. In the face of colleagues’ knowledge requests, individuals are more likely to conceal or retain complex, implicit or difficult to encode knowledge than clear and understandable knowledge (Connelly and Zweig, 2015; Pan et al., 2018; Hernaus et al., 2019). Specifically, the acquisition of this kind of knowledge often takes a lot of individual time and energy. As a result, there is a greater acquisition cost, so individuals are more inclined to retain this knowledge. (2) Personal factors mainly include individual perception and other psychological factors. The negative emotion of employees at work is the main factor that determines whether they share knowledge or not (Zhao and Xia, 2019). For example, Ali et al. (2020) and He et al. (2020) discussed the formation mechanism of knowledge withholding from the perspective of perceived threat of individual job security. Malik et al. (2019) used questionnaire surveys and linear regression method, to believe that professional commitment in individual factors would inhibit employees’ perception of the positive correlation between organizational politics and knowledge withholding. (3) At the level of organizational environment, scholars have discussed the effects of organizational culture, organizational management policies and systems on individual knowledge withholding. Serenko and Bontis (2016) believe that organizational management systems and policies can significantly promote employees’ knowledge withholding. At the same time, organizational unfair treatment will promote employees to actively retain knowledge and enhance the level of knowledge withholding among employees (Abubakar et al., 2019; Jahanzeb et al., 2020).

As far as online virtual communities are concerned, the existing research and the leading research focus on the two factors of the individual and the environment. (1) In terms of personal factors, Shen et al. (2019) constructed a SEM model based on the perspective of secondary control. They believed that the knowledge contribution self-efficacy (predictive control) and social attribution (substitution control) of users have both positive effects on knowledge withholding. Based on the neutralization theory, Sun et al. (2015) showed that users in the brand community would use neutralization technology to rationalize their norm-bias behaviors. That is, individual denial of responsibility, denial of harm, etc., will promote the generation of knowledge withholding. (2) In terms of environmental atmosphere, Alashoor et al. (2017) pointed out that users will be more willing to publish information when they feel that the community provides strong privacy and security protection.

In summary, the research results on knowledge withholding have enriched the basic theory and research content of this field, but there are still some gaps. (1) At present, most scholars’ discussions on knowledge withholding are based on offline entity organizations. Compared with offline organizations, there is a lack of social connection between users in the online virtual environments, and scientific research users have a unique professional background. This will lead to more vital territorial awareness of knowledge (Xu, 2011). (2) Most of the current researches is based on a single perspective of promoting or inhibiting the phenomenon of knowledge withholding. However, in the online organization of virtual communities, the withholding of user knowledge is often the result of the interaction of individual users, knowledge objects, and the organizational environment (Zhang and Zhang, 2017). (3) Mostly existing studies on factors affecting knowledge withholding use regression or structural equation method (SEM). This causal relationship model has certain limitations, including difficulty in interactive influence and non-linear analysis, inability to deal with missing data, and poor fit (Zhao and Wan, 2003). Through self-learning ability to model the complex relationship between input and output, the Neural network model (ANN) can make up for the problem of the fitting accuracy of structural equations to non-linear data.

In order to enrich the research content in the field of online knowledge withholding. This study focuses on the comprehensive mechanism of influencing factors of online community knowledge withholding from the dual perspectives of enabling and inhibiting. So as to systematically and completely reveal the formation mechanism of user knowledge withholding. To answer the following research questions:

Q1: What is the difference in the mechanism of individual knowledge withholding between online virtual academic communities and offline organizations?
Q2: From the perspective of enabling and inhibiting, how do knowledge, individual, and environment affect online user knowledge withholding?
Q3: Are there differences in the impact of different factors on user knowledge withholding?
Q4: Can the combination of traditional statistical methods and ANN methods improve the fitting degree of the model?

In response to the above problems, this research uses virtual academic communities as the research object. Based on sociological theories and psychological ownership theories, it divides the social attributes of knowledge, personal factors, and organizational environment from the two dimensions of enabling and disabling. The hybrid research method combining structural equation and artificial neural network (ANN) is used to construct and verify the model. First, the structural equation model is used to determine the causal relationship model between knowledge withholding and its influencing factors. Secondly, the connection mode between neurons in each layer is customized by using the non-linear characteristics and self-learning ability of neural network. Then, the coefficients of the model are updated, which not only solves the linear hypothesis of the structural equation, but also makes the weight update of the neural network model reasonable.

## Theory and Hypothesis

Although a single type of influencing factor will promote the generation of user knowledge withholding, the current research ignores the comprehensive impact of different influencing factors on knowledge withholding. Moreover, in the selection of influencing factors, compared with the natural attribute of knowledge and individual self-perception, the social attribute of knowledge and personal perception under the influence of others have not attracted enough attention of scholars. At the same time, privacy protection is one of the research hotspots from the online community. Scholars mainly focus on private information involving user assets and personal health, such as business community and medical community. In addition, although reciprocity in the organizational environment has been studied in offline organizations, it lacks empirical support from online organizations.

Based on psychological ownership theory, this study takes psychological knowledge ownership and knowledge power in the attribute of knowledge society (Jin et al., 2019), subjective norms of knowledge withholding and professional commitment in personal factors, community reciprocity, and community privacy protection in the attribute of organizational environment as antecedents. According to the difference in the characteristics of the influencing factors, they are summarized into two dimensions: enabling and inhibiting (the former is mainly used to promote user knowledge withholding positively, and the latter is to interfere user knowledge withholding negatively). We are exploring the combined causality between influencing factors and user knowledge withholding in virtual academic communities through a dual perspective.

### Enabling Dimension

#### Knowledge Psychological Ownership and Knowledge Withholding

Based on the psychological ownership theory, the ownership perceived by an individual is expressed as a sense of possession of the object. The individual with this feeling regards the object as an extension of himself, which will affect his attitude toward the object (Pierce et al., 2001). If individual ownership exists in an organization, knowledge privatization will occur in the process of knowledge transfer. In an organizational environment, individuals have a sense of belonging to tangible and intangible things (Pierce and Jussila, 2011). When individuals have a sense of ownership of their knowledge, it may lead to conflict consciousness and psychological conflict when sharing knowledge (Jonathan et al., 2021). From the research of relevant scholars, when a person spends a certain amount of time and energy to obtain knowledge that others do not master, it is easy to produce his goods. Then he is willing to retain knowledge and refuses to share it with others. Therefore, the following hypotheses are proposed in this study:

H1: Virtual academic community users’ knowledge psychological ownership positively affects knowledge withholding.

#### Knowledge Power and Knowledge Psychological Ownership

When knowledge owners have differences in quantity, quality, and type of knowledge, knowledge surplus will often occur, and then knowledge-power will appear. Individuals with unique knowledge can make the organization or other individuals attached to them, thus bringing extraordinary power and improving their influence ability in the organization (Wang et al., 2020). Although individuals share knowledge, their knowledge reserves will not decrease. However, with the mastery of this knowledge by other individuals, the individual’s knowledge power will be weakened, which will enhance the loss of their knowledge power. When knowledge owners have the fear or fear of losing knowledge power, their willingness to share knowledge with others will also be reduced (Li and Liu, 2014). Therefore, individuals tend to regard knowledge power as their own unique privilege, and this desire to possess the target is the expression of territoriality (Peng, 2012). According to the principle of territoriality, the more an individual attaches importance to knowledge power, the more likely it will be to produce the individual psychological ownership of knowledge. Therefore, the following hypotheses are proposed in this study:

H2: Knowledge power has a positive impact on the knowledge psychological ownership of users in the virtual academic community.

#### Subjective Norms of Knowledge Withholding and Knowledge Psychological Ownership

According to social influence theory, an individual’s attitude, belief, and behavior will be influenced by people or groups around him. Subjective norms refer to the perception that users think people who have an important impact on them want to express specific behaviors (Ajzen, 2002). It reflects how individuals are influenced by important people (Schofield, 1974). According to the above view, subjective norms of knowledge withholding indicate whether an individual believes that others’ knowledge withholding in the network is correct. Generally speaking, the norms of an individual for a specific behavior are mainly formed by observing the behavior of other important individuals around him. When vital people around individuals (such as tutors or classmates, bosses or friends) encourage them to actively communicate and share knowledge with users in the community by using the virtual academic community around them, it will be considered inappropriate to regard knowledge as their private goods and refuse to share it with others (Ajzen and Fishbein, 1980). On the contrary, when most people around an individual choose to only acquire knowledge without making contributions in the virtual academic community, an individual will tend to regard knowledge as his goods and refuse to share knowledge (Ifinedo, 2012). Thus, there is a significant correlation between subjective norms and individual attitudes and behavioral intentions. Therefore, the following hypotheses are proposed in this study:

H3: Subjective norms of knowledge withholding have a positive impact on users’ knowledge psychological ownership in virtual academic communities.

#### Professional Commitment and Knowledge Psychological Ownership

Individuals who highly recognize and love their majors care more about professional development and their contributions to their majors than those around them. Such recognition of their majors and efforts is called professional commitment (Lian et al., 2005). Individuals with a high sense of professional commitment tend to care more about the development of their profession (Tuan et al., 2020). Based on their enthusiasm and satisfaction for their work, they believe that professional knowledge should be spread to more people, so they actively communicate and share knowledge with the questioners in the face of consultation from other users in the virtual community. Such individuals are more willing to find a partner with a high degree of professional commitment through communication and the exchange of professional knowledge (Buhari et al., 2020). Therefore, the following hypotheses are proposed in this study:

H4: Professional commitment negatively affects the knowledge psychological ownership of users in virtual academic communities.

### Inhibiting Dimension

#### Community Reciprocity and Knowledge Withholding

Reciprocity, as a social norm, can be defined as a community atmosphere in which users share knowledge in an organization and hope to be rewarded when they need help in the future (Wang, 2019). Based on the social exchange theory, positive reciprocity emphasizes that individuals respond to perceived friendly requests. When the individual faces the positive request initiated by the other party, it will produce a more positive reciprocal response (Mitchell et al., 2012). Therefore, a good interpersonal relationship needs to be formed through continuous reciprocal exchange between both sides. Frequent reciprocal communication between users can meet the needs of both sides, thus creating a sense of reliance and mutual, and promoting continuous knowledge communication between the two sides. When individuals find other users’ knowledge withholding behaviors, they imitate others’ behaviors and conceal or refuse to share knowledge with others (Cropanzano and Mitchell, 2005; Anupam et al., 2018). For the virtual community, users communicate in an anonymous environment, and text has become the only way to get along. When users have a positive expectation for the behavior of community users and have obtained the knowledge help of others, they will have a sense of knowledge reciprocity to the community. Thus, affected by the community environment, actively participate in the knowledge exchange of community users. Establishing a mechanism of mutual trust between users will promote knowledge sharing among users in the community and reduce the willingness of users to retain knowledge. To this end, this paper proposes the following hypotheses:

H5: Community reciprocity negatively affects the knowledge withholding of users in virtual academic communities.

#### Community Privacy Protection and Knowledge Withholding

Based on social cognitive theory, individual behavior, cognition, and environment can interact to produce dynamic influence. The perspective of behavior covers not only an individual’s actual action, but also the intention to make action, which can accurately predict and react to individual behavior. Korzaan and Boswell (2016) found that network information privacy is an essential factor affecting users’ personal information protection in the network. At the same time, users who pay high attention to personal information will actively take measures to reduce information exposure. They are unwilling or avoid information sharing and communication on the Internet in order to reduce the possibility of their personal information exposure. Dwyer et al. (2007) said that some users avoid personal information leakage by reducing their online use time and expressing their views and opinions on network social media. According to Osatuyi (2015), when users are under the protection of personal information, they will be in a diving state in the virtual community and only acquire knowledge while reducing knowledge sharing in the community, thus resulting in knowledge withholding behavior. Therefore, the following hypotheses are proposed in this study:

H6: Community privacy protection negatively affects the knowledge withholding of users in virtual academic communities.

**FIGURE 1:**
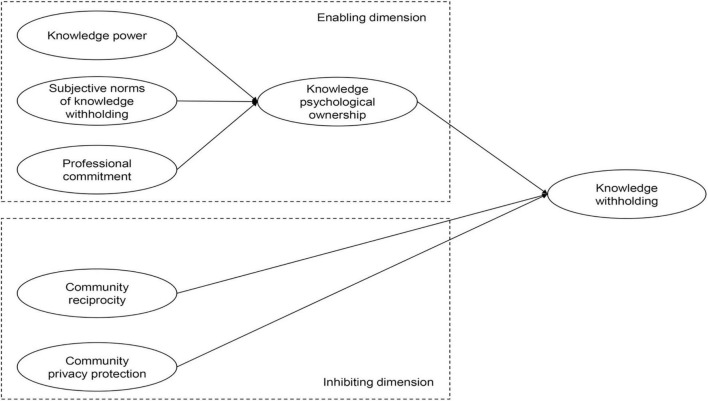
Influencing factor model of user knowledge withholding in virtual academic community.

Based on the above hypothesis, this paper establishes a model of influencing factors of user knowledge withholding in the virtual academic communities, as shown in Figure 1.

## Methodology

### Sample and Procedures

The research data were collected by issuing network questionnaires. To ensure the authenticity and reliability of the data, the author first collected the information of the current mainstream virtual academic community in China. After the investigation and evaluation of the user scale and community activity, the author selected Xiaomuchong (Science and Engineering Section), CSDN, Jingguanzhijia, Kuaiji community, Dingxiangyuan, and Aiai medical website as the research objects from the three fields of science and engineering, management and medicine.

The experimental process lasted about 3 months from design, implementation to completion. To ensure the stability and accuracy of the measurement of research variables, pre-investigation was carried out at the initial stage of the experiment. A small-scale questionnaire collection was posted in Xiaomuchong, Jingguanzhijia, and Dingxiangyuan. A total of 150 questionnaires were distributed, 128 valid questionnaires were recovered, and the recovery rate was 85.3%. According to the results of the reliability and validity test of the questionnaire, the questionnaire was sorted and revised, and the items with a factor load less than 0.5 were deleted. This stage lasted 30 days. Then, the questionnaire was officially distributed and collected. The author posted relevant questionnaire filling posts in the reward section or help section of the above six communities, and set a specific reward gold coin. At the same time, some users were randomly selected by private letter to invite them to participate in the questionnaire filling. Each ID can only be filled in once to avoid the repeatability of the questionnaire. 720 questionnaires were collected, and 616 valid questionnaires were obtained after excluding invalid questionnaires, including 211 in the science and technology community, 228 in the management community, and 177 in the medical community. The effective recovery rate of the questionnaire was 86%.

In the valid sample, the gender distribution of the respondents is balanced, and the proportion of men and women is 49.4 and 50.64%, respectively. The age distribution of the respondents is mainly 21–30 years old. Most of them have bachelor’s or master’s degrees, accounting for about 80%. At the same time, 67.5% of the respondents belong to school students or workers in scientific research institutions, indicating that most of the users of the virtual academic community are middle-aged and young intellectuals. Their daily work and study are closely related to professional knowledge, based on the basic information of users. The investigation believes that while maintaining the number of visits and activity of such people, we can appropriately drain and refresh the scientific research practitioners or enterprise knowledge employees over the age of 35, to expand the scope of use of the virtual academic community and inject new vitality into the community. See Table 1 for the usage of users’ virtual community. About 73% of users’ virtual community usage time is concentrated within 3 years. At the same time, in the virtual academic community, more than 80% of users post and reply between 0 and 11 posts per month, with an average interaction of up to 3 days. It can be seen that in the virtual academic community, the overall proportion of truly active users in the community is not high, and a considerable number of users are still in the stage of collecting knowledge rather than contributing knowledge. The basic information of user-specific community use is shown in Table 1.

**TABLE 1:** Basic information of virtual community users.

Measurement variables	Option	Percentage (%)
	High school and below	2.3
	Junior college	7.1
Education	Bachelor	43.5
	Master	35.4
	Ph.D. and above	11.7
	Business worker	16.2
	Workers of government agencies	12.5
Profession	Student	37
	University or research institute member	30.5
	Other	3.8
Community use time	Within 1 year	32.5
	1–3 years	40.4
	More than 3 years	27.1
Average monthly posts	0–5 posts	50.3
	6–11 posts	34.3
	12 posts and above	15.4
Average monthly replies	0–5 replies	42.4
	6–11 replies	40.9
	12 replies and above	16.7

### Measures

Since knowledge withholding is caused by individual subjective feelings, it is suitable to use self-report (Connelly and Zweig, 2015). This study was conducted by questionnaire. The questionnaire mainly includes two parts: the first is the basic information of the respondents and community use, and the second is the measurement of knowledge withholding and its influencing factors. The variable items in the questionnaire are collected in the form of the Likert 7 scale. 1–7 respectively represents the interval from “very disagree” to “very agree.” The variable items in the questionnaire adopt the mature scale that has been studied and used. The back-translation procedure is implemented for the relevant foreign literature hierarchy, that is, first translate the English scale into Chinese, and then back translate it into English according to the Chinese scale, compare the differences between the two and make corresponding modifications. At the same time, considering the differences in language and environment, this study modifies some items and wording of the questionnaire based on comprehensive expert opinions. For example, in the object of knowledge withholding, change “I contribute fewer knowledge to the virtual community than I know” to “I will make less efforts in knowledge contribution than I know and can answer.”

The specific scale design is as follows: (1) In terms of knowledge attributes, knowledge power (KP) is measured by Kankanhalli et al. (2005), including three items: “knowledge is the source of personal rights and status, especially my unique knowledge.” The psychological ownership of knowledge (KPO) is adapted from the scale of Jarvenpaa and Staples (2001), including “I think the knowledge accumulated in my study and work belongs to me.” (2) In terms of personal perception, professional commitment (PC) is adapted from Teng et al. (2009) scale, including three items such as “I care about the future development of my field of study.” The subjective norm of knowledge withholding (SN) is adapted from the scale of Wu (2020), including three items: “my superiors (tutors) and colleagues (classmates) think I should retain my knowledge in the virtual community.” (3) In terms of community environment, Community reciprocity (CR) adopts the scale of Lin and Huang (2010), including “when I share knowledge in the community, others will actively answer my questions.” Community privacy protection (CPP) adopts the scale adapted from Kordzadeh and Warren (2017), including three items such as “when I answer questions in the community, I am not worried about personal information being leaked.” (4) Knowledge withholding (KW) adopts the scale prepared by Tsay et al. (2014) and others, including four items such as “contributing knowledge to community members is not the main concern of my participation in the community.”

### Two-Stage Hybrid Research Method

Based on the idea of the parallel hybrid method, the traditional statistical method and machine learning method are not a substitute relationship, but can complement each other. Conventional statistical methods can play a significant role in causal identification, inference analysis, and dimension simplification. Compared with conventional statistical methods, the machine learning method is more flexible in parameter modeling. It does not give specific assumptions to the relationship between data and variables, but directly learns and explores the statistical relationship between features and variables according to the objective function. Therefore, statistical analysis and machine learning are combined (Hong and Wang, 2021). Make them support each other and cooperate effectively, triangular verify the research objectives, and further interpret the theoretical model.

**FIGURE 2:**
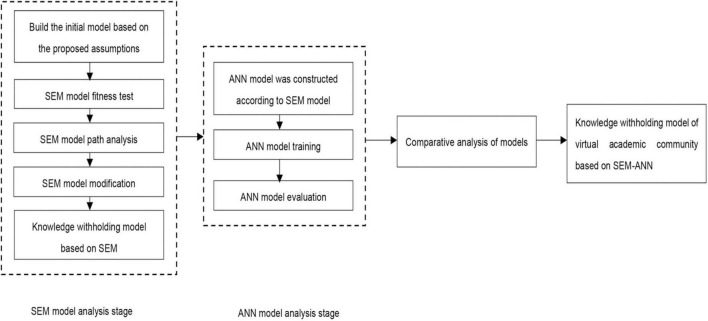
Structural equation method-artificial neural network (SEM-ANN) model flow chart.

Therefore, a two-stage hybrid research method combining structural equation analysis and neural network is proposed in this paper. In the first stage, the primary purpose of structural equation analysis is to demonstrate the path hypothesis between various antecedent variables and knowledge withholding. In the second stage, the primary purpose of neural network analysis is to update the path coefficient of structural equation and improve the fair degree of model. The specific analysis process is shown in Figure 2.

(1) Stage 1: Structural Equation stage

According to the relevant theoretical basis, the research constructs the structural equation model of the above influencing factors and knowledge withholding. After model verification, path analysis and model correction are carried out. Finally, a causal relationship model between influencing factors and knowledge retention is obtained.

(2) Stage 2: Neural Network Analysis stage

The SEM causality model is transformed into a multi-layer feedforward neural network topology spanning the connection (Zhao and Wan, 2003). Ultimately, the path weight output by the SEM model is used as the initial weight of the ANN model, and the self-learning ability and non-linear mapping ability of the neural network are used to fit and update the model.

## Results

### Evaluation of the Measurement Model

This article uses SPSS and AMOS software to test the reliability and validity of the scale (Li et al., 2021). (1) Reliability test. It can be seen from Table 2 that composite reliability (CR) of each latent variable is between 0.785 and 0.878. According to Wu (2010) and Namra et al. (2021), the composite reliability threshold is 0.7. The CR values of the research variables are higher than the minimum requirement level of 0.7, indicating that the scale has good reliability. (2) Construct validity. When the KMO value of the scale is more significant than 0.5, and the *P*-value of the Bartlett sphere test is less than 0.05, the data is considered to have good construct validity (Yu and Liu, 2018). From Table 2, the overall KMO value of the questionnaire is 0.905, and the *P*-value of the Bartlett sphere test is 0, confirming the measurement model’s reliability. (3) Convergent validity. Convergent validity is reflected by AVE, which is used to measure whether the measurement items can fully describe the variables (Fornell and Larcker, 1981). It can be seen from Table 2 that the average variance variation AVE of each latent variable is greater than the minimum standard of 0.5 (Bagozzi and Yi, 1988), which shows that the scale has good convergence validity. (4) Discriminant validity. According to Fornell and Larcker (1981) criterion, it can be seen from Table 2 that the square root of the AVE of each latent variable is larger than the correlation coefficient between the latent variable and other latent variables (Awan et al., 2021), which means that the latent variables of the scale have a certain degree of discrimination. The discriminative validity of the data is ideal. Finally, we use the Hamann single-test to test the common method bias of the data. The first principal component is 38.5% without rotation 50% less than the boundary standard (Podsakoff et al., 2003), which does not explain most variables. This means that common method bias is not severe enough for statistical study.

**TABLE 2:** Questionnaire reliability and validity test.

	Latent variables	1	2	3	4	5	6	7
(1)	KP	0.806						
(2)	KPO	0.739	0.714					
(3)	PC	0.346	0.384	0.801				
(4)	SN	0.769	0.707	0.395	0.751			
(5)	CR	−0.418	−0.450	−0.061	−0.327	0.837		
(6)	CPP	−0.283	−0.349	−0.148	−0.250	0.275	0.837	
(7)	KW	0.762	0.713	0.303	0.711	−0.567	−0.468	0.727
	AVE	0.662	0.551	0.645	0.573	0.706	0.701	0.707
	CR	0.852	0.785	0.845	0.800	0.878	0.875	0.878

	KMO	0.905						
	Bartlett *P*	0.000						
	Cumulative variance	38.5%						

### Structural Equation Method -Artificial Neural Network Model Analysis Process

#### Structural Equation Method Model Analysis

With the aid of AMOS software, the virtual academic community knowledge withholding path model is constructed based on the theoretical model and tested. The test results of the essential fitness of the model are obtained. The RMSEA value of the absolute fitness index is 0.094, and the GFI value is 0.797. The matching indexes IFI, TLI, and CFI are 0.836, 0.814, and 0.836, respectively, which are all greater than 0.7 (Hu and Bentler, 1999). The reduced fit index PGFI and PNFI are 0.638 and 0.729, respectively, which are both greater than 0.6 (Mulaik et al., 1989; Byrne, 1998). So, the model is acceptable.

In terms of the hypothesis testing of the model, it can be seen from Table 3 that except for hypothesis H4, other hypotheses are supported. From the perspective of enabling dimension, knowledge psychological ownership (β = 0. 853, *P* < 0.001) has a significant positive impact on knowledge withholding, and hypothesis H1 is true. Knowledge power (β = 0.860, *P* < 0.001), subjective norm of knowledge withholding (β = 0.507, *P* < 0.001) had a significant positive impact on knowledge psychological ownership, and H2 and H3 were assumed to be true. Professional commitment (β = 0.048, *P* = 0.124) has no significant effect on knowledge psychological ownership, and H4 is not tenable. From the perspective of inhibition, community privacy protection (β = −0.335, *P* < 0.001), community reciprocity (β = −0.401, *P* < 0.001) has a significant negative impact on knowledge withholding. It is assumed that H5 and H6 are true.

**TABLE 3:** Structural equation path test results.

		Estimate		*S.E*.	*P*
KP	→	KPO	0.860	0.032	***
SN	→	KPO	0.507	0.036	***
PC	→	KPO	0.048	0.028	0.124
KPO	→	KW	0.853	0.066	***
CR	→	KW	−0.335	0.028	***
CPP	→	KW	−0.401	0.029	***

n = 616; ***p < 0.001.

Because the negative impact of professional commitment on knowledge psychological ownership did not pass the significance test, the action path of professional commitment on knowledge psychological ownership was deleted. It is found that professional commitment is affected by four dimensions, including emotional commitment, ideal commitment, normative commitment, and continuing commitment. The four dimensions of individual professional commitment are based on professional identity, personal identity, and social identity. The deviation of identity at any stage will impact on individual professional commitment (Zhang, 2015). In the virtual academic community, some individuals with a high sense of continuing commitment, believe that professional knowledge is closely related to future work. They insist that sharing knowledge with others will weaken their employment advantages to a certain extent. Therefore, the inhibitory effect of such individuals’ professional commitment on individual psychological ownership of knowledge is not apparent, which affects the significance of the whole path.

The results of the modified model test and path test are shown in Table 4. From the test results, the fitness test of the modified model is higher than the acceptable level, which is higher than most of the index values before correction. Moreover, the *P*-values of all path tests were less than 0.001, which significantly passed the path test.

**TABLE 4:** Modified model test.

Absolute fitness index			Value added fitness index		Parsimony fit index	
RMSE	GFI	IFI	TLI	CFI	PGFI	PNFI
0.082	0.804	0.839	0.813	0.838	0.621	0.710

**FIGURE 3:**
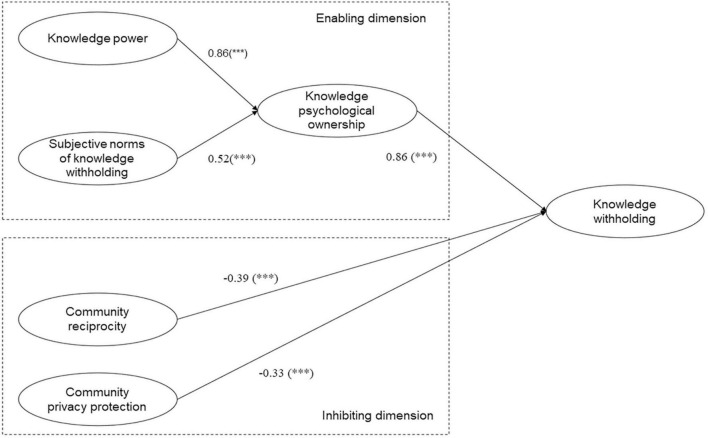
Structural equation method (SEM) path map of knowledge withholding in virtual academic community. ***P < 0.001.

Based on the above index values, it can be considered that the modified SEM causality model has good fitting validity. Finally, the SEM causality model is obtained, as shown in Figure 3.

#### ANM Model Analysis

**FIGURE 4:**
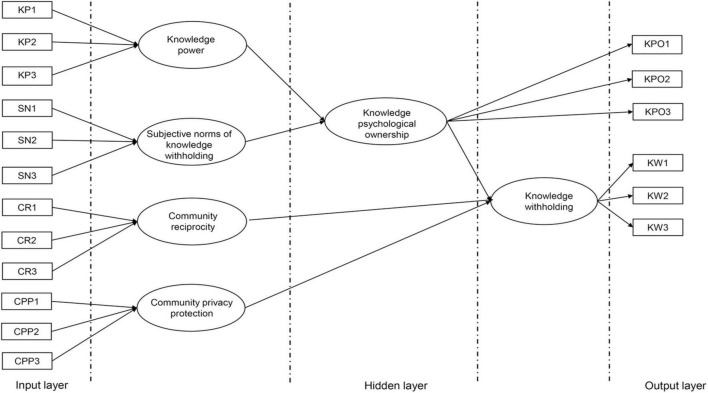
Structural equation method-artificial neural network structure.

According to the above structural equation model, PyTorch is used to build a non-fully connected artificial neural network topology (shown in Figure 4). The exogenous measurement variables of knowledge power, subjective norms of knowledge withholding, community reciprocity, and community privacy protection are used as input data. The number of hidden layers and neurons is determined by potential variables and the connection mode between potential variables. In this paper, the number of hidden layers is set to 2. The two neurons of knowledge power and subjective norms of knowledge withholding integrate the output results as the neuron input of knowledge psychological ownership. The three neurons of knowledge psychological ownership, community reciprocity, and community privacy protection integrate the output results as the input of knowledge withholding neurons. The number of neurons in the output layer is determined by the number of measurement variables of knowledge psychological ownership and knowledge withholding. Then the path coefficient of SEM model in Figure 3 is input as the initial weight of connection between neurons in each layer.

Before data input, first, carry out standardization processing, and then set the training function and training parameters. The training data set and test data set are randomly divided according to the ratio of 8:2. After the training convergence of the sample data, compare the training effects of different parameters, and finally determine that the activation function is set as the sigmoid function. The optimizer selects Adam for parameter optimization, the learning rate is set to 0.015, and the maximum number of iterations allowed is 1,500. Calculate the RMSE and *R*^2^ output by the model, and the results are shown in Table 5.

**TABLE 5:** Structural equation method-artificial neural network (SEM-ANN) evaluation index of knowledge withholding.

Measurement index	Knowledge psychological ownership			Knowledge withholding		
	KPO1	KPO2	KPO3	KW1	KW2	KW3
RMSE	0.105	0.108	0.113	0.100	0.111	0.092
*R* ^2^	32.0%	35.3%	64.4%	67.3%	48.8%	63.0%

It can be seen from Table 5 that the root mean square error of SEM-ANN model fitting is about 0.1, indicating that the model has achieved a good convergence effect after 1,500 iterations. At the same time, the *R*^2^ of each output index is up to 67.3%, with an average of about 52%. In the field of Sociology and user behavior research, a goodness of fit greater than 25% indicates that the variable is interpreted to a high extent (Zhang, 2004; Zhang et al., 2018). Therefore, the fitting effect of the model is good after training.

#### Comparative Analysis

The fitting degree of SEM model and SEM-ANN model is further compared through the judgment coefficient. The comparison results are shown in Table 6 below.

**TABLE 6:** Comparison between SEM model and SEM-ANN model.

Model	Knowledge psychological ownership			Knowledge withholding		
	KPO1	KPO2	KPO3	KW1	KW2	KW3
SEM	31.7%	33.8%	58.4%	52.5%	38.0%	39.8%
SEM-ANN	32.0%	35.3%	64.4%	67.3%	48.8%	63.0%

It can be seen from Table 6 that the goodness of fit of output variables is higher than that of the SEM model, and the interpretation degree of KPO3, KW1, KW2, and KW3 is significantly increased by about 10%. The results show that after the causal relationship between variables is determined by the structural equation model, the neural network is introduced to update the path coefficient, which not only improves the interpretability of the model, but also improves the goodness of fit of the structural equation model.

The path coefficients and neuron connection weights of the SEM and the SEM-ANN models are normalized, and the updated coefficients are shown in Table 7. Compared with the effect of different influencing factors on user knowledge withholding in the SEM model, the inhibitory effect of inhibitory factors fitted by the SEM-ANN model on individual knowledge withholding is significantly higher than that of enabling factors. From the side, it reflects that if the community can enhance the sense of mutual benefit of community knowledge and create a sense of mutual help for everyone. At the same time, managers strengthen the protection of community privacy and make users of virtual academic community feel a sense of information security. Then, to a certain extent, it will reduce the impact of users’ own psychological ownership of knowledge on their knowledge withholding, and promote users to be willing to exchange knowledge.

**TABLE 7:** Comparison of knowledge withholding path coefficients between SEM and SEM-ANN.

Path	SEM	SEM-ANN
KP → KPO	0.62	0.67
SN → KPO	0.38	0.33
KPO → KW	0.54	0.50
CR → KW	−0.25	−0.32
CPP → KW	−0.21	−0.18

## Discussion and Conclusion

This study takes the users of the virtual academic community as the research object, and tries to construct the SEM-ANN knowledge withholding influencing factor model from the new perspective of enabling and inhibiting. We profoundly discuss the comprehensive influence mechanism of knowledge social attributes, personal factors and organizational environment on knowledge withholding in the online virtual academic communities. The research fills the gaps in the online field of knowledge withholding research. At the same time, it enriches the research dimensions of knowledge withholding influencing factors, and provides a reference for the governance of knowledge withholding phenomena in the online communities. From this research, the following key findings can be obtained:

Firstly, compared with offline entity organizations, knowledge withholding is more likely to occur in online virtual academic communities. Through literature review, we find that due to the weak contractual relationship between individuals in online space and the lack of material incentives to participate in knowledge sharing. Therefore, people tend to retain all knowledge in online communities (Cranefield et al., 2015). Especially in the virtual academic community with knowledge sharing and exchange as the core, combined with the professional background of such users, scientific researchers often need to write patents and experimental schemes. Higher patents and experimental results mean that individuals have a higher knowledge surplus than other users. And then generate more knowledge power (Wang et al., 2020) to maintain its driving force for continuous innovation. Therefore, the awareness of knowledge protection of scientific researchers will be more vital. At the same time, the online virtual community environment will aggravate the psychological insecurity of researchers and make the nature of hiding knowledge more obvious (Wang, 2019).

Secondly, the results of SEM path test from a dual perspective show that: (1) In the enabling dimension, the impact of professional commitment on users’ psychological ownership of knowledge is not significant. Knowledge power is the main reason to promote users to form psychological ownership of knowledge, and then affect users’ knowledge withholding. Some studies have found that individuals with a high sense of professional commitment are willing to seek partners with the same belief to explore their knowledge and obtain common knowledge (Shu and Zhai, 2019). However, the relationship between users’ professional commitment and psychological ownership of knowledge in virtual academic community is not significant. The result is inconsistent with existing studies. This is because some users in the virtual academic community have a high sense of continuous commitment to professional commitment. They take professional knowledge as a livelihood and believe that sharing knowledge will lose their own value. The reason why knowledge power is the main influencing factor of users’ psychological ownership of knowledge is that the essence of virtual academic community is based on a large number of knowledge resources. The unequal knowledge resources among users will naturally produce knowledge resource dependence, and knowledge resource dependence will inevitably lead to the generation of knowledge power (Ma, 2005). Unbalanced knowledge power will contribute to the phenomenon of power erosion (Gupta and Govindarajan, 2000), which further promotes the generation of user knowledge withholding. (2) In the inhibition dimension, reciprocity and community privacy protection have a significant negative impact on user knowledge withholding. Community reciprocity plays a vital role in restraining community knowledge withholding and promoting the stable development of the community. Especially in the virtual academic community, individuals often give back the knowledge benefits obtained by actively participating in knowledge sharing activities under the effect of community reciprocity (Yin et al., 2021). In addition to community reciprocity, community privacy protection will also have a certain inhibitory effect on knowledge withholding. Nowadays, due to the imperfect personal privacy data protection mechanism, the trust relationship, privacy sensitivity and the balance between perceived risks and benefits felt by users are superimposed, resulting in low willingness of users to exchange and share knowledge (Liu and Wang, 2018; Zhang Y. D. et al., 2021). Therefore, the key to resolve the risk of user privacy data disclosure and reduce user awareness is to establish a stable privacy protection system and formulate an efficient community privacy protection mechanism.

Thirdly, through the relevant results of the SEM-ANN model, it is found that the combined inhibitory effect of community environment on knowledge withholding in the enabling dimension is strengthened. In contrast, the promoting effect of knowledge psychological ownership on user knowledge withholding in the enabling dimension is weakened. Organizational environment is the external environment in which individuals engage in corporate activities, including social organization working environment and physical working environment. Social organization working environment includes incentive guarantee of organization series and organizational atmosphere factors. The organizational environment has an important impact on individual actions in the organization. At the same time, compared to the effects of other factors on individual actions, individuals in knowledge-based organizations have a more significant effect on the perception of organizational environment (Ni et al., 2015). Generally speaking, the key to the sustainable development of a virtual academic community lies in improving of community knowledge quality. Community order and incentive atmosphere are often used as the driving force of community knowledge output. This positive knowledge co- construction environment will continuously stimulate users’ desire for knowledge exchange and reduce users’ diving mentality (Li et al., 2020). Therefore, the strengthening of the inhibitory effect from organizational environment factors will weaken the promoting effect of individual subjective consciousness on knowledge withholding to a certain extent.

Finally, the combination of SEM and ANN can improve the fitting effect of the model. Comparing the final path coefficients of SEM and SEM-ANN models, in the SEM-ANN model, the impact of knowledge psychological ownership on knowledge withholding is significantly improved, and the inhibitory effect of community environment on knowledge withholding is also enhanced. By combining neural network with structural equation, the interpretability of neural network model is improved, and the non-linear fitting ability of neural network is used to make up for the deficiency of structural equation.

## Theoretical and Practical Implications

### Theoretical Implications

(1) Different from positive knowledge behaviors such as knowledge sharing. This research takes knowledge withholding, an anti-production knowledge behavior, as an entry point. From the perspectives of enabling and inhibiting, it analyzes the influencing mechanism of the factors affecting the withholding of user knowledge in the virtual academic community. To a certain extent, it has enriched the research results in this field. At present, the research on virtual community at home and abroad mainly focuses on positive knowledge management behaviors such as knowledge sharing. The research on negative knowledge management behavior such as knowledge withholding is not rich. And most of the existing studies are based on a single perspective analysis, ignoring the synergy and mutual check and balance mechanism of the two opposing factors of enabling and restraining. This research incorporates sociological theories and psychological theories into a research framework. From the two dimensions of enabling and inhibiting, the factors affecting user knowledge withholding are divided and considered comprehensively. Through a comprehensive analysis of the influence mechanism of the phenomenon of knowledge withholding in virtual academic communities, the research conclusions are more convincing and stable.
(2) For the first time, knowledge, individual and environment factors are integrated into the research scope of influencing factors of knowledge withholding, which supplements the research scene and content in this field. Based on the existing research, most researchers only observe the influence of independent elements and ignore the combined effect of different influencing factors, especially online communities. The research focuses on the impact of personal and environmental factors on user knowledge withholding. This study discusses the internal causes of knowledge withholding in virtual academic community from the perspective of knowledge, individual and environment, to make the achievements in this field richer and more representative.
(3) A two-stage hybrid research method combining structural equation analysis and neural network is proposed. This method can integrate the statistical method with the conclusion of machine learning in order to supplement and improve the research results. Structural equation analysis aims to verify the causal path hypothesis between influencing factors and knowledge withholding. Further, the validated structural equation model is transformed into the corresponding neural network model. This method improves the interpretability of the model and updates the coefficients at the same time. It is a verification and supplement to the results of structural equation. The application of this hybrid method provides a new idea and method for subsequent related research.

### Practical Implications

This research provides some targeted suggestions for the management of virtual academic communities:

(1) Actively take a variety of effective measures to reduce users’ awareness of knowledge privacy and knowledge power. The more substantial users’ awareness of knowledge power, the stronger their awareness of knowledge psychological ownership. When users have a hunch that they will lose their unique value and privilege in the community after knowledge exchange, it will promote knowledge privacy. In the daily management of virtual academic communities, community managers can actively promote the concept of knowledge sharing in the community, increase the rewards for users who actively share knowledge, and make knowledge contributors feel encouraged and recognized. Guide users to transition from “my” knowledge to “our” knowledge, giving equal reputation and gold coins to respondents at different levels. At the same time, it can increase the traceability function of the respondents to the original answers, create a good community atmosphere of respecting originality and knowledge, and reduce the respondents’ sense of loss of knowledge power.
(2) Give full consideration to the reasonable demands of users for the community environment, and create a mutually beneficial and friendly academic atmosphere and a community environment that respects personal privacy. The community can design a sign representing the reciprocity level on the ID or avatar according to the number of active answers to attract users to actively participate in knowledge exchange. At the same time, the function option of anonymous answer is provided to strengthen the protection of users’ private information. Not only that, the community also needs to curb the emergence of pop-up information outside the platform, encourages users to report privacy related issues and punish misconduct.
(3) Weigh the impact of enabling and inhibiting dimensions, and flexibly adjust community management measures. The phenomenon of knowledge withholding in the virtual academic communities is driven by the combined action of enabling and inhibiting dimensions. The change of users’ psychological cognition needs not only time, but also the subtle influence of their environment. Therefore, community managers can enhance the impact of inhibiting factors on knowledge withholding. The inhibition effect of enabling dimension factors on knowledge withholding is reduced. At the same time, additional experience rewards are given to users who actively reply, to enhance the construction of a sense of community safety and interactive atmosphere, so as to establish a good community service environment, reduce users’ subjective knowledge withholding from the side, and promote the sustainable development of virtual academic community.

## Limitations and Further Research

This study has the following limitations. Firstly, the data collected in the form of questionnaire has a certain subjectivity, which cannot entirely objectively describe the situation of user knowledge withholding. The objective information of the user page is not effectively obtained, such as the number of followers, the number of posts, user level, etc. Secondly, this study takes China’s virtual academic community as the research object. Due to China’s unique social culture (Zhang H. Q. et al., 2021), which may have an additional impact on the phenomenon of individual knowledge withholding. Finally, although the virtual academic community is a representative kind of community with knowledge exchange as the core, and the research on the phenomenon of user knowledge withholding has certain reference significance for the virtual community, the conclusion is based on a single type of virtual community must have some limitations.

Future research work can consider combing subjective and objective methods to obtain more accurate user knowledge withholding information. For example, collect subjective data through questionnaires and crawl the objective data from the user’s home page to collect data on user knowledge withholding. At the same time, future research should integrate more cultural and influencing factors to enrich the research results. For example, conduct surveys on virtual communities in China and other countries. Meanwhile, we can compare and analyze the influencing factors and mechanism of community knowledge withholding under different cultural backgrounds. Finally, future research should use the existing research framework to test other types of communities, such as friendship communities and virtual brand communities, so as to further expand the research field of knowledge withholding online organizations.

## Data Availability Statement

The raw data supporting the conclusions of this article will be made available by the authors, without under reservation.

## Ethics Statement

Ethical review and approval was not required for the current study in accordance with the local legislation and institutional requirements. The patients/participants provided their written informed consent to participate in this study. Relevant data was anonymously collected from the respondents based on the principle of voluntary participation.

## Author Contributions

CL determined the topic selection, ideas, structure and research methods of the manuscript, and guide the revision of the manuscript. WL conducted questionnaire survey, manuscript writing, chart drawing, and manuscript modification. Both authors contributed to the article and approved the submitted version.

## Conflict of Interest

The authors declare that the research was conducted in the absence of any commercial or financial relationships that could be construed as a potential conflict of interest.

## Publisher’s Note

All claims expressed in this article are solely those of the authors and do not necessarily represent those of their affiliated organizations, or those of the publisher, the editors and the reviewers. Any product that may be evaluated in this article, or claim that may be made by its manufacturer, is not guaranteed or endorsed by the publisher.
