# Supplementary material for: Analysis on the Influence Path of User Knowledge Withholding in Virtual Academic Community – Based on Structural Equation Method-Artificial Neural Network Model
Source: Front Psychol. 2022 Feb 7;13:764857. doi: 10.3389/fpsyg.2022.764857 (PMC8860021; doi:10.3389/fpsyg.2022.764857)
Supplement: Supplementary file 1 [file Table_1.DOCX]

**Questionnaire on knowledge withholding in virtual academic community**

This is a questionnaire about user knowledge withholding in virtual academic communities (such as Xiaomuchong, CSDN, etc.), and the respondents are friends who have used or participated in such communities. There are no standard answers or correct answers to the questions in the body of the questionnaire. Please answer according to your actual situation or true feelings. The questionnaire is only used for academic research, will not be used for any commercial purpose, and will not disclose your privacy. Finally, thank you very much for taking the time out of your busy schedule to fill in the questionnaire.

**Part I basic information of the preparer**

1. What kind of virtual academic community do you usually use

A.Jingguanzhijia B. Xiaomuchong C. CSDN D. Ding Xiangyuan E. Kuaiji community

F. Aiai medical website

2. Your gender:

A. Male B. Female

3. Your age group:

A. 20 years and younger B. 21-25 C. 26-30 D. 30-35 E. 36 years and older

4. Your education:

A. High school and below B. College C. Bachelor D. Master E. Doctor and above

5. Your occupation:

A. Business worker B. Workers of government agencies C. Student

D. University or research institute member E. others

6.How long has it been since you first used the community：

A. Within 1 year B. 1-3 years C. More than 3 years

7.Your average monthly posting frequency is：

A. 0-5 articles / month B. 6-11 articles / month C.12 articles and above / month

8. Your average monthly reply frequency is：

A. 0-5 articles / month B. 6-11 articles / month C.12 articles and above / month

**Part II Questionnaire body**

Please judge whether the following statements are in line with your situation. 1 represents complete disagreement; 2. The representative strongly disagrees; 3. The representative disagrees; 4 represents uncertainty; 5. The representative agrees; 6. The representative agrees very much; The representative fully agrees. The larger the number, the more you agree. Please click on the appropriate level for the following questions according to the degree of agreement or disagreement.

| Observation variable | Questionnaire items | 1 | 2 | 3 | 4 | 5 | 6 | 7 |
| --- | --- | --- | --- | --- | --- | --- | --- | --- |
| Knowledge power | Knowledge is the source of personal rights and status, especially my unique knowledge |  |  |  |  |  |  |  |
|  | When I open or share knowledge, I will feel that I lose the knowledge that can make me stand out |  |  |  |  |  |  |  |
|  | When I open or share my knowledge, I will feel that I have lost my unique value in the community |  |  |  |  |  |  |  |
| Knowledge  psychological ownership | I think the knowledge accumulated in my study and work belongs to me |  |  |  |  |  |  |  |
|  | I think the knowledge I bring to study and work belongs to me personally |  |  |  |  |  |  |  |
|  | I don't think my knowledge can be used by others |  |  |  |  |  |  |  |
| Professional commitment | I am loyal to my major |  |  |  |  |  |  |  |
|  | I care about the future development of my field of study |  |  |  |  |  |  |  |
|  | Compared with other majors, I love my major more |  |  |  |  |  |  |  |
| Subjective norms of knowledge withholding | My superiors (tutors) and colleagues (classmates) think I should keep my knowledge in the virtual community |  |  |  |  |  |  |  |
|  | Most people who have learning value for me have retained their knowledge in the virtual academic community |  |  |  |  |  |  |  |
|  | Generally speaking, when I disagree with my superiors (tutors), I will give priority to my superiors (tutors) |  |  |  |  |  |  |  |
| Community reciprocity | When I share knowledge in the community, others will actively answer my questions |  |  |  |  |  |  |  |
|  | When sharing knowledge in the community, I also hope to get the knowledge I need in the future |  |  |  |  |  |  |  |
|  | When sharing knowledge in the community, I hope others can respond positively to me when I need knowledge |  |  |  |  |  |  |  |
| Community privacy protection | My community attaches importance to the protection of users' personal information |  |  |  |  |  |  |  |
|  | When I answer questions in the community, I am not worried about personal information being leaked |  |  |  |  |  |  |  |
|  | I don't worry about disclosing information in the community because it won't be used in a way I haven't met |  |  |  |  |  |  |  |
| Knowledge withholding | I generally try to avoid contributing knowledge in virtual communities |  |  |  |  |  |  |  |
|  | I will devote less effort to knowledge contribution than I know and can answer |  |  |  |  |  |  |  |
|  | Contributing knowledge to community members is not my main focus in participating in the community |  |  |  |  |  |  |  |
